# A review on the evolution of methods for intestinal *in vitro* organ culture and its application in veterinary science

**DOI:** 10.14202/vetworld.2023.347-356

**Published:** 2023-02-21

**Authors:** Barbara Ribeiro de Souza Cortez, Roberto Maurício Carvalho Guedes

**Affiliations:** 1 Department of Veterinary Clinic and Surgery, Universidade Federal de Minas Gerais, Belo Horizonte, Brazil; 2 Department of Large Animal Clinical Sciences, University of Saskatchewan, Saskatoon, Canada

**Keywords:** enteropathogens, explants culture, intestinal pathogens, swine colon, ussing chamber

## Abstract

Different techniques have been reported in studies of intestinal *in vitro* organ culture (IVOC). A robust compilation of all available methods is lacking in the literature, making it difficult to choose a method that corresponds to the study’s demands. In this review, readers can assess the most available methods, allowing them to evaluate which is more suitable for their purposes and requirements. A simplified view of culturing intestinal explants is presented, highlighting the approachability of IVOC. Relevant findings from diverse veterinarian studies, where explants played a major role, as well as the technique used in each, are described to illustrate its applications. Finally, the strengths and limitations of the innovative intestinal IVOC methods are discussed. This review provides a collection of methods for intestinal explant culture and their possible applications in veterinary research. In this way, it aims to broaden access to IVOC techniques and aid decision-making regarding the best suited for a study’s purposes.

## Introduction

The intestinal mucosa is one of the main surfaces for interactions between individuals and the outside world, and it is responsible for dealing with microorganisms, pathogens, and potentially harmful agents. The complex arrangement of specialized tissues interacts in countless ways; therefore, a sophisticated model that synthesizes these myriads of processes during pathologic and physiologic states is critical for veterinary and human medical research. Cell models possess limited clonal populations and fail to reproduce an organism’s *in vivo* architecture. However, proper access to the intestinal mucosa for *in vivo* sampling is anatomically, technically, and ethically challenging. Despite this, animal gastrointestinal models pose considerable laborious maintenance and intensive logistics and are often financially unapproachable [1].

Consequently, intestinal *in vitro* organ culture (IVOC) models have been developed and increasingly used to investigate intestinal physiology, pathogenic mechanisms, and pharmacological interactions, with the advantage of maintaining cellular variety and differentiation simultaneously with tissue interactions. When it comes to biology, the word *explant* translates to a piece of tissue or organ that has been removed from an organism and cultivated, maintaining its cells alive and able to divide. Culturing explants allows researchers to perform interspecies studies under reproducible conditions, harvest several tissues from a single donor, reduce the number of animals needed, and control and sensibly manipulate the surrounding conditions, which is ideal for a detailed exploration of factors controlling migration and cellular differentiation in several intestinal compartments [2]. Intestinal explant-based models offer a more controlled environment for experimental manipulation than *in vivo* models. Thus, new intestinal IVOC techniques represent a robust alternative to the demands presented by veterinarian researchers worldwide.

Different research groups have developed countless techniques over the decades, each possessing unique methodologies and specific research applications. The diversity in methodologies and complexity lead to difficulty in evaluating these applications of intestinal IVOC techniques separately in new studies. This review aims to summarize the methodologies involved in relevant achievements in veterinary research and disclose their techniques. Accordingly, this review aims to broaden access to these techniques and facilitate the researchers’ decision-making on IVOC methodologies in veterinary studies.

## Intestinal IVOC Techniques and its Achievements in Veterinary Research

There are various methods for culturing intestinal explants. The complexity level of each technique is often related to the desired outcome type. For example, in most cases, specialized equipment is required to maintain explants for longer periods. In addition, if the measured response is related to living organisms in the gut, the technique will necessarily be more sophisticated because no microbiome suppressors can be used; therefore, tissue degradation is exacerbated. In this way, this review will be divided into categories, beginning with simpler methods and scaling up for methods requiring specialized apparatus to maintain intestinal explants.

## Methods Suited for Embryonic Tissues Cultivation

Methods to maintain embryonic tissue survival were the first to be developed. Beck and Thomson [3] and Fischer [4] cultured embryonic tissues from chickens and obtained varying degrees of differentiation *in vitro*. The first attempt to culture mature organs was accomplished by Trowell in 1954 [5] with an enhanced apparatus, culture medium, and methodology to cultivate several types of tissues for up to 9 days sustaining a satisfactory morphological state. Although intestinal culture was not performed, it was considered problematic because of bacterial contaminants inherent to this organ.

**Figure-1:**
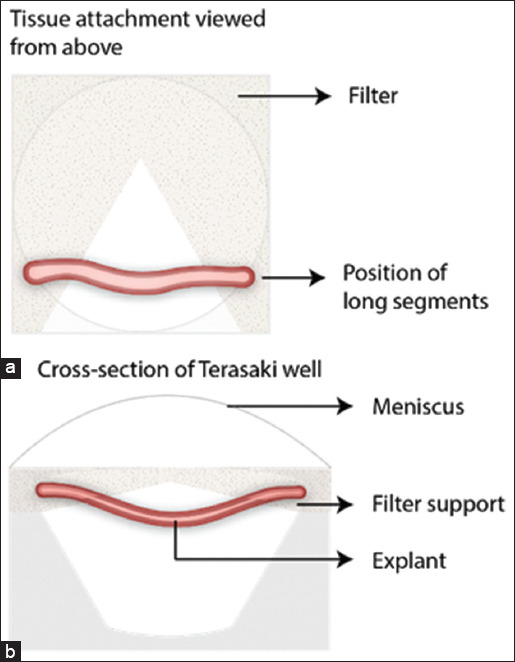
Schematic figure of explants under catenary culture [6]. (a) The tubular segments of the embryonic intestine have their edges fixed on a piece of paper filter cut in a “V” shape. (b) This setting is then placed inside a well of a Terasaki plate which is filled with culture media.

In 1999, Hearn *et al*. [6] developed a “catenary culture” method. Its name is related to the catenary form acquired by the wires when attached to its edges. Intestinal explants were fixed at the ends of a “V” shape cut made into 3 mm^2^ pieces of 0.45 μm Millipore paper (Figure-1) [7].

This method allows cultivation with morphological maintenance for up to 10 days, which is only possible due to the embryonic and aseptic origin of the intestine. The purpose of this technique was to access enteric neural crest-derived cell migration in embryos, maintaining the proper formation of other structures and functions, such as the muscle layer, goblet cells, and gut motility. The peculiar positioning of explants is required to maintain the organ’s tubular shape with the serosal side facing outwards since eversion and deformation of explants have been observed by the previous authors who performed submerged culture. A subsequent study performed by the same research group using this technique indicated the likely role of a glial-derived neurotrophic factor in enteric neural crest-derived cell migration [7], which was further confirmed by Goto *et al*. [8] in 2013.

## Methods Using Simple Apparatus

In 1969, Browning and Trier [9] detailed a modified version of Trowell’s technique, which allowed the cultivation of human small intestinal mucosal biopsies for up to 24 h, maintaining morphological and functional features. Instead of the apparatus previously proposed, these researchers used a circular plastic dish containing a smaller well in its center, where the medium was added. A triangular metal mesh was docked on top of the inner dish such that its bottom touched the surface of the medium lying beneath. The intestinal explants were placed above the mesh, with the basolateral side facing downward and the mucosal side facing upward. In this arrangement, the explants constantly absorb the medium through capillarity. The dishes were then incubated at 37°C with 95% O_2_ and 5% CO_2_ for 12–24 h. Explants were exposed to thymidine-H and micellar solution to study cell proliferation and the ability of enterocytes to absorb micellar fat. It was shown that, after 24 h of cultivation, crypt cells had evidence of DNA synthesis and enterocyte function of absorbing lipids, and the synthesis of triglycerides was conserved.

**Figure-2:**
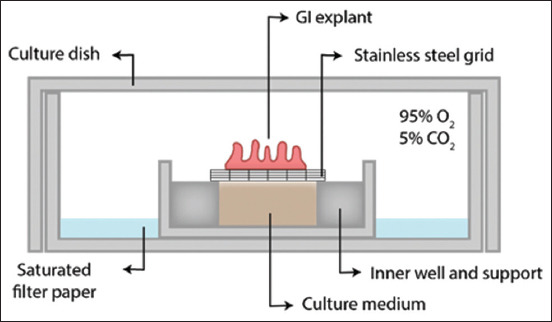
Diagram of Eastwood and Trier explant setting [2]. The apparatus was composed of a culture dish with an inner well, allowing the creation of a dead space filled with water-imbibed filter paper. A stainless steel grid was placed on top of the central well such that the culture medium underneath touched the bottom of the grid and provided nutrients through capillarity for the explant. The entire setting was placed inside an incubator with a 95% O_2_ and 5% CO_2_ atmosphere.

Adaptations for the cultivation of human large intestinal mucosal biopsies were evaluated by Eastwood and Trier [10]. The apparatus used was the same metal grid coupled into the small well centered inside the culture dish, as described previously, with the addition of a sponge saturated with water around the central well for better atmospheric hydration (Figure-2) [2]. The authors found that explants maintained signs of epithelial cell proliferation and preserved their morphological features for at least 24 h.

In 1978, Shamsuddin *et al*. [11] proposed a method for the cultivation of colonic tissue stripped from the serosal layer. Each fragment measured 5 mm^2^ and was placed at the edge of large Petri dishes (60 mm diameter) with a small amount of CMRL 1066 medium (2 mL) (Sigma-Aldrich, USA). When placed on a rocker platform, this setting allowed the explants to be bathed 50% of the time. The plates were incubated in a chamber with 95% O_2_ and 5% CO_2_ at 37°C. The medium was renewed after 24 h and then every 2 days. The authors claimed that they achieved a cultivation time of 20 days. In 1991, Nietfeld *et al*. [12] achieved better results using RPMI 1640 cell culture medium (Sigma-Aldrich) instead of CMRL 1066 and obtained a 48 h culture.

In 1994, Zhu *et al*. [13] improved this method by placing explants on top of biopsy sponges humidified with the culture medium underneath. Recently, Gaudio *et al*. [14] performed a pharmacological study of fosfomycin using swine jejunal explants infected with *Lawsonia intracellularis* aiming to evaluate the concentration of this antibiotic inside enterocytes using Zhu’s technique with modifications. Briefly, jejunal fragments measuring 3 cm in length were collected, opened on the mesenteric border, placed on Petri dishes, and washed twice with physiological saline solution through shaking. Then, circular sections of 1.3 cm^2^ weighting 0.1 g were placed above the sponges with the mucosa facing up. Each sponge was submerged into a well of a 6-well culture plate containing Dulbecco’s modified Eagle’s medium (DMEM) supplemented with L-glutamine, high glucose, and F-12 nutrient mix from Gibco. The explant preparation was completed in <1 h after euthanasia. Subsequently, 580 μg/mL of fosfomycin was added to explants with 500 μL of the bacteria, obtained from the commercial vaccine Enterisol Ileitis^®^ (Boehringer Ingelheim, Germany) in half of them. The plates were maintained on a shaker at 37°C for up to 48 h. This study concluded that explants were histologically better conserved after 24 h of cultivation. Moreover, the presence or absence of *L. intracellularis* did not impair the intracellular concentrations of fosfomycin. Using this method, Girard *et al*. [15] aided in the comprehension of the mechanism used by enteropathogenic *Escherichia coli* to cause disease in swine after observing the attaching and effacing phenotype in ileal explants. In addition, they reinforced the finding that *E. coli* subtypes containing the intimin virulence factor present more effective adherence to enterocytes.

Kolf-Clauw *et al*. [16] adapted this method and applied it to study mycotoxicity in the swine intestine. The researchers used the same medium without ascorbic acid and contained 100 U/mL penicillin, 100 μg/mL streptomycin, and 50 μg/mL gentamicin. Their study sought to validate this explant model for mycotoxin assays through the histological evaluation of exposed explants. They concluded that this approach is sensitive to the effects of food contaminants on the intestinal epithelium. Moreover, additional studies on the effects of mycotoxins on the intestine have been conducted. For instance, da Silva *et al*. [17, 18] reported that phytic acid, a natural antioxidant, reduced histological changes and oxidative stress in swine jejunal explants exposed to the mycotoxins fumonisin and deoxynivalenol (DON). Another study by the same group in 2019 [19] characterized histological changes in jejunal explants from piglets exposed to chito-oligosaccharide (COS), a potential substitute for in-feed antibiotics. The study found that the histological score, villus height, crypt depth, and villus: crypt ratio between the COS-treated and mock-treated groups were statistically equal at treatment levels from 0.025 to 0.15 mg/mL. This finding supports the potential use of this substance at low dosages without causing harm to the intestinal mucosa of piglets; however, the ability to protect the animal against challenge was not explored. Using the same technique, Maidana *et al*. [20] tested the ability of metabolites from *Lactobacillus plantarum* to reduce DON toxicity in piglet jejunal explants.

Duarte *et al*. [21] explored the protective effects of antimycotoxin additives (AMAs) against deoxinivalenol exposure on intestinal explants using broiler chicken jejuni. This study also emphasized histopathological analysis, looking at parameters such as villi number and height, crypt diameter, villi height/crypt diameter ratio, enterocyte height and nucleus size, cytoplasmic vacuolization of enterocytes, microvilli integrity, and immunostaining of caspase-3. The authors described no differences between AMA-treated and untreated explants prior to DON exposure, indicating the inefficacy of the products tested. In this way, it was possible to indicate the likely response of a substance before an *in vivo* study to reduce animal use.

In 2012, Tsilingiri *et al*. [22] introduced an innovative method to add stimuli to the explants. Instead of adding bacteria, food additives, or medicines to the culture medium, this technique polarizes the stimuli by confining it to a cylinder adhered to the intestinal mucosa. This apparatus was composed of a Petri dish with an inner well at its center. Inside the inner well, the culture medium was added, and a triangular metal grid was sustained by its edges, similar to the setting of Eastwood and Trier [10], as shown in Figure-2. The intestinal fragment striped from the serosal layer was placed on top of the mesh, with the serosal side facing downwards and the mucosal side facing upward. The differential is the cylindrical structure placed on top of the explant so that the desired substance can be placed inside it, and the stimulus remains confined at the mucosal surface.

The study performed by Tsilingiri *et al*. [22] showed the interaction of probiotics and a pathogenic species of *Salmonella* by comparing both polarized and non-polarized methods. The results showed that in the polarized model, a better representation of *in vivo* contamination occurred, in addition to greater conservation of the mucus layer. This layer is a natural protective barrier, and the attachment of several pathogenic agents to enterocytes is hampered or assisted by altering its mucin composition.

**Figure-3:**
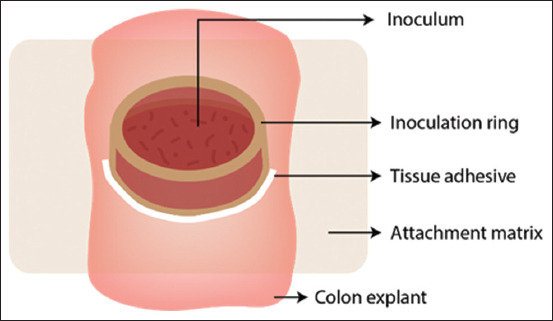
Diagram of explant setting [24]. The intestinal fragment striped from the serosal layer is attached with an agar block - the attachment matrix - which allows nutrients from the culture media around it to permeate and nourish the explant. Above the explant, an inoculation ring is attached with tissue glue then the desired stimulus can be placed and confined to the mucosal surface. The entire setting is set inside a hyperoxide chamber at 37°C.

A few years later, Costa *et al*. [23] developed a technique optimized for porcine colon explant cultures. It consists of sectioning a segment of 8 cm from the apex of the spiral colon, which is placed into a vial containing refrigerated Dulbecco’s phosphate-buffered saline without calcium or magnesium and supplemented with antibiotics. The segment was then opened in its mesenteric border, and the serosal layer was mechanically removed. Afterward, sections of 1.5 cm^2^ were obtained and placed into 1% agar blocks, arranged inside Petri dishes containing 8 mL of keratinocyte basal medium supplemented with 1.5 mM calcium and antibiotics. Polypropylene cylinders were attached to explants using tissue glue to polarize the stimulus (100 μL of inoculum containing *Brachyspira hampsonii* was added in this study). Subsequently, the plates were incubated in hyperoxic chambers containing 99% O_2_ and 1% CO_2_ for 8 h (Figure-3) [24].

**Figure-4:**
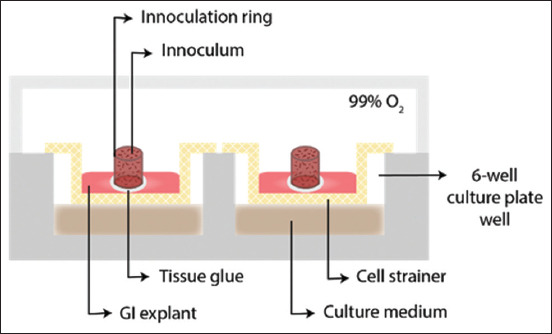
Representation of porcine colon explant culture upgrade [1]. Each plate contains 6 explants independently cultured with polarized stimulus. The setting is composed of a 6-well culture plate, each with a layer of culture media underneath the cell strainer touching its bottom. One explant per well is placed with the mucosal side facing upwards and the serosal side facing downwards. Then an inoculation ring is attached with tissue glue, and the inoculum is placed inside. Several plates can be placed inside hyperoxide chambers and undergo culture at 37°C incubators.

Researchers have aimed to demonstrate the pathological changes found in pigs with swine dysentery by measuring the metabolic response of explants exposed to the causative agent and their histopathological evaluation. The study concluded that this pathogenic species of *Brachyspira* is capable of inducing necrosis *in vitro*, probably through a mechanism mediated by interleukin (IL)-1α and reactive oxygen species. In 2018, the same authors published a book chapter displaying the above-described technique with an upgrade of using a cell strainer to contain the explant, replacing the agar block (Figure-4) [1]. As a result, the method became much easier and more approachable to apply without any loss in explant survival and morphological preservation, achieving 5 days under culture [1].

In 2017, Udden *et al*. [25] developed a method for colon culture that lasts for 24 h and is suitable for studying the microbial host defense response. The authors obtained 1 cm^2^ colon pieces and then transferred them into 100 μm cell strainers placed inside 6-well plates and added 5 mL of DMEM/F12 medium with antibiotics. In this method, colon segments were submerged. The plates were incubated at 37°C in 95% air and 5% CO_2_. Every 2 h, 3 times, the medium was renewed using an antibiotic-free medium. Then, colon explants were transferred into a well of a 12-well culture plate with DMEM/F12 and 5% fetal bovine serum (FBS) and cultivated for another 12 h. In this study, explants were stimulated with IL-1 beta, IL-18, or left untreated and then subjected to a reverse transcription-polymerase chain reaction for IL and antimicrobial peptide gene expression measurements. The supernatant was collected and incubated with *E. coli* to determine whether explants with induced inflammation were better at releasing antimicrobial peptides in the supernatant. The authors found that stimulated explants expressed higher levels of antimicrobial peptides and ILs and produced a supernatant more capable of killing bacteria than the control. Thus, this is an additional valuable method for studying intestinal antimicrobial immune responses.

Similar to Udden *et al*. [25], who created a submerged culture of intestinal explants instead of maintaining the explant outside the culture media, Bareiss *et al*. [26] proposed a three-dimensional culture model. According to the authors, this method allows colon explants to survive for up to 2 weeks. The technique consists of cleaning colon fragments with HBSS containing penicillin, streptomycin, and metronidazole, followed by opening at the mesenteric border, slicing 2 mm^2^ explants, and transferring them into Millipore membranes of 0.45 μm pore size. Then, 1 mL of 4-(2-hydroxyethyl)-1-piperazineethanesulfonic acid buffered DMEM/F12 medium containing 10% horse adult serum, penicillin, streptomycin, L-glutamine, insulin/transferrin/selenite mix, albumin, hydrocortisone, glucagon, 3, 3’, 5’-triiodo-L-thyronine, ascorbate-2-phosphate, linoleic acid, estradiol, and keratinocyte growth factor was added. The plates containing six explants were placed in humidified incubators with 5% CO_2_ and 37°C atmosphere, and the medium was renewed every 2 days. Researchers infected explants with wild-type and a knockout strain of *Candida albicans* and verified that the yeast causes similar effects when infecting explants compared to *in vivo* infection, concluding that this *in vitro* model is suitable for gut infection experiments.

Baydoun *et al*. [27] released an adapted enhanced version, in which differences consisted of using 12 mm^2^ explants and filtering the medium prior to use with a 0.22 μm filter. Researchers have used severely combined immunodeficient mice, which seemingly contributed to extended explant survival since they reached at least 4 weeks of cultivation. This study highlights the potential use of this technique for *Cryptosporidium parvum* infection assays, as well as further drug screening for this parasite, in addition to a technique that ensures a relatively long cultivation period.

**Figure-5:**
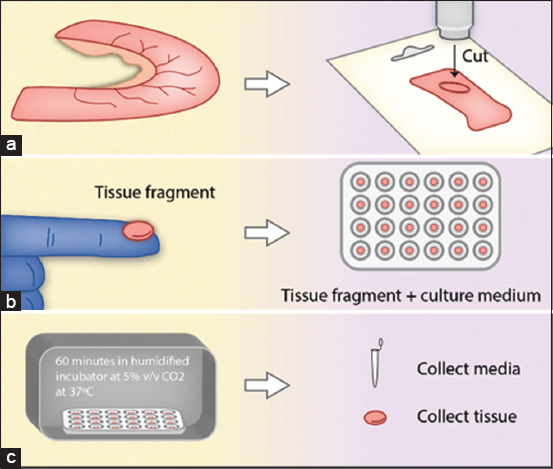
Demonstration of segment model setup [29], (a) The intestinal segment is cleaned and placed on culture media. (b) The segment is cut open on its mesenteric border, placed (mucosa facing down) on a refrigerated platform, and cut with a biopsy punch. (c) The biopsied fragments are obtained and individually placed on 24-well culture plates containing culture media.

Verhoeckx *et al*. [28] described a method referred to as a segment model [29] that also uses the submerged approach. It involves obtaining intestinal fragments and placing them in ice-cold Krebs-Ring Bicarbonate buffer with 95% O_2_ and 5% CO_2_ until processing. The segment was then cut open in its mesenteric border, and the serosal layer was carefully stripped out. Circular fragments were sectioned with 8 mm biopsy punches, which were placed inside 24-well culture plates filled with 500 μL buffer. Plates were placed inside humidified incubators containing 5% CO_2_ v/v and cultivated for 1.5 h (Figure-5) [29].

This method is used to study the effects of nutrients and compounds on gut metabolism and intestinal hormone release, which can be analyzed from either the supernatant or explants. Voortman *et al*. [30] used this technique to evaluate the effects of long-and short-chain fatty acids on the release of gastrointestinal hormones. The authors found increased levels of glucagon-like peptides 1 and 2 when explants received both fatty acids. This research highlights the relevance of the approach for studying satiety compounds, which might be relevant for animal health and production.

In 2016, Barato *et al*. [31] described a method for culturing fish intestinal explants that used the same principles as previous submerged techniques. Anterior intestine portions of 4 cm each obtained from tilapias (*Oreochromis* spp.) were placed in 140 mm^2^ Petri dishes, 20 mL of high glucose DMEM was added, and incubated at 28°C. Next, the intestinal lumen was rinsed with DMEM three times through a syringe application, and the explants were transferred into 24-well plates containing DMEM with 10% FBS. The plates were incubated inside chambers in a controlled atmosphere (5% CO_2_ and 80% humidity) at 28°C under mild agitation for up to 40 min. The authors evaluated the adherence ability of encapsulated and unencapsulated *Streptococcus agalactiae* to the intestinal epithelium. The research concluded that the capsule impairs bacterial adhesion during the first steps of infection and that an acidic environment favors the adhesion of encapsulated bacteria. In 2019, Vásquez-Machado *et al*. [32] applied the same technique and revealed that the bacteria discarded its capsule before attachment to the apical border of the intestinal villi. After entering the enterocyte cytoplasm, the bacteria continue to replicate and subsequently achieve basolateral portions, where they invade the lamina propria and travel through microcirculation, without an evident inflammatory response or caliciform cell reaction.

In 2019, Kothari and Rajagopalan [33] released a protocol for isolating rat intestinal explants for *in vitro* culture, which is suitable for toxicology and metabolism studies. The technique relies on tissue oxygenation through the culture media, the polarization of stimuli by the inversion of intestinal fragments, and the stabilization of the tissue on a manufactured Matrigel™-(Corning Inc., USA, cat. no. 354234) coated polydimethylsiloxane rod. Briefly, intestinal segments were placed on cold oxygenated culture media DMEM with sodium bicarbonate (0.37% w/v), FBS (10% v/v), penicillin-streptomycin (200 U/mL Pen-200 μg/mL Strep), gentamicin (50 μg/mL), porcine insulin (0.5 U/mL), epidermal growth factor (20 ng/mL), glucagon (14.28 ng/mL), hydrocortisone (1.65 μg/mL), and oxygenated for 45 min/liter. Then, with the aid of a grooved rod, the fragment was inverted, rinsed, and cut into 10 mm pieces. Intestinal fragments were moved into Millicell^®^ (EMD Millipore, USA, cat. no. MCSP12H48) 12-well culture plate hanging inserts coated with collagen gel and covered with 3 mL of oxygenated intestinal medium. After preparation, the explants were incubated at 37°C in a humidified incubator with 10% CO_2_ for up to 24 h. The authors affirmed that other animals could be used as a source of intestinal explants following this technique as long as the diameter of the intestine remains unchanged (4 mm).

## Methods Using Specialized Apparatus

### Ussing chamber

**Figure-6:**
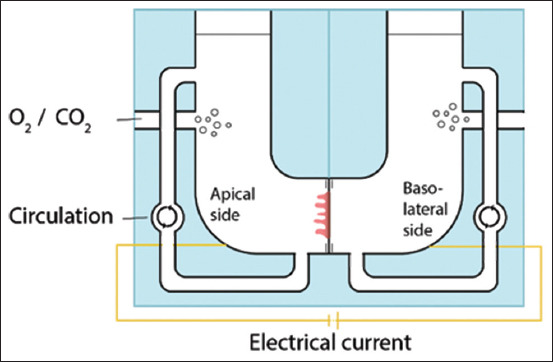
Representation of an Ussing chamber mounted with a fragment of the intestine [36]. The apical and basolateral sides have separated chambers that can receive different stimuli. A supply of oxygen and carbonic gas is constantly pumped into the media, which is recirculated inside each chamber. The device has an underlying electrical current used to measure trans-epithelial electrical resistance and detect ionic differences related to Na+/H+ and Cl-/HCO_3_- membrane exchangers.

In 1951, Ussing and Zerahn [34] created the Ussing chamber, a device capable of measuring the ongoing ion transport within cultivated tissues. In this study, a fragment of frog skin was cultivated for 5 h. The breakthrough achieved here was due to the elucidation of processes such as electrogenic Cl^-^ secretion, electroneutral NaCl absorption, and electrogenic Na^+^-coupled glucose absorption in diverse contexts in the intestinal mucosa. Concomitantly, it was the first technique that allowed stimulus separation of the basolateral from apical surfaces (Figure-6) [34, 35], considering its capability to measure ion transport. Ussing chambers can be used for a broad range of purposes on intestinal explants, such as micronutrient transportation evaluation, inflammation, toxicological, and hormonal studies, the pathogenesis of intestinal agents, and enzymatic activity measurement [36]. An example of its use is a study conducted in 2020, which aided the understanding of swine dysentery pathogenesis. Researchers have observed that the bacteria responsible for this disease can inhibit Na^+^ absorption in the colon of pigs, causing an osmotic imbalance that leads to diarrhea [37].

Gustafsson *et al*. [38] proposed the use of a perfusion chamber, similar to the Ussing chamber, to culture colonic and ileal explants. Researchers can measure mucus layer penetration, thickness, and spontaneous mucus growth through this approach. Immediately after obtaining intestinal portions, they were washed with cold Krebs’ solution oxygenated with 95% O_2_ and 5% CO_2_, enriched with 116.0 mM NaCl, 1.3 mM CaCl_2_, 3.6 mM KCl, 1.4 mM KH_2_PO_4_, 23.0 mM NaHCO_3_, and 1.2 mM μgSO_4_ at pH 7.4. The muscular layer was mechanically removed, and the explants were mounted into perfusion chambers for mucus thickness measurement or image chambers for mucus penetrability studies. Their study found that spontaneous growth of the mucus layer is conserved in colonic explants, and the proportions of the inner and outer mucus layers were similar to those reported *in vivo*.

Importantly, due to the short cultivation time provided by this mechanism on intestinal tissues (up to 150 min), animals were required to be exposed to certain stimuli, mainly pathogenic agents, prior to euthanasia. Therefore, the advantage of reducing animals within an experimental group and diminishing costs regarding facilities and animal care is not feasible in cases in which researchers seek a late response from a given stimulus. Another limitation is the low throughput of the technique, which requires one chamber setting for each explant.

Therefore, new devices are being developed to overcome this limitation. Horizontal chambers are an alternative to the classical Ussing chamber, where tissues are cultivated vertically. For example, two main devices have been designed for this purpose. First, the NaviCyte Horizontal Ussing System supports 6 chambers simultaneously, maintaining inlet and outlet pumps for liquid recirculation. Second, the in TESTine™ system from TNO-Netherlands Organization for Applied Scientific Research (Zeist, The Netherlands) allows the simultaneous cultivation of 24–96 explants using simplified smaller chambers. The latter system does not support recirculation; instead, the apparatus must be placed on a rocker platform so liquids can be agitated inside the chambers [36].

### PigutIVM

A method for intestinal explant cultivation, PigutIVM, has been published by Fleury *et al*. [39]. The authors optimized a bioreactor for the culture of intestinal explants and prioritized the maintenance of intestinal microbiota. This method allows explant survival for at least 28 days and is suitable for studying probiotics, antibiotics, feed ingredients, and other subjects related to the microbiota. The biggest disadvantage is the requirement of a specific bioreactor and the time-consuming task of learning how to operate it.

## Peristalsis Recording Methods

In 1958, Bülbring *et al*. [40] described a method for recording peristalsis in isolated intestinal sections. This technique relies on a device that produces electric waves induced through the intestine and is registered in a volume recorder. Neither this nor later published studies have evaluated the viability of the tested intestinal segment using histopathology or any other method since the goal here concerned the muscle layer’s electrical responsiveness. As explants, for definition, must maintain cells alive and able to divide, the method for recording intestinal peristalsis is unsuitable for this review. It is a valuable method for studying drugs and evaluating their effects on longitudinal muscle contractions, intraluminal pressure, and volume of fluid expelled through the intestinal segment.

It is relevant to mention a mighty, although expensive, option developed by Minekus *et al*. [41] in association with The Netherlands Organization for Applied Scientific Research (TNO). The TNO gastrointestinal model was optimized to control peristalsis and physiological fluids to measure the absorption of nutrients and drugs. This device uses synthetic tissues instead of explants and is not a suitable subject for great detailed mention in this review.

## Organoids

**Figure-7:**
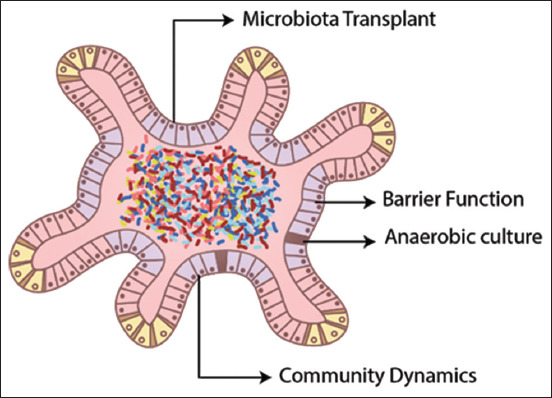
Schematic drawing of an organoid [44]. The external layer is composed of all the different cell types of the intestinal mucosa, where the pale pink cells represent intestinal crypts and the blue cells represent the intestinal surface. The interior of the enteroid, in this case, has received a microbiota transplant, so its lumen contains a population of bacteria.

Organoids, or enteroids, when the stem cells originate from enterocytes, have the advantage of using a single animal to obtain an enteroid culture that can be used for an undefined time, increasing statistical representativity. The main limitation is the spherical form that they assume, which occludes the lumen and makes it difficult to expose the mucosa to pathogens or substances, in addition to being a technique of poor yield [42]. A method to invert polarity was developed, allowing the mucosa surface to face outside; however, its use is still insipient [43]. In addition, a technique of microinjection that produces high output and allows the addition of microbiota to enteroids has been developed recently (Figure-7) [44, 45]. They are a more suitable model to evaluate the regulation and renewal of epithelium, but they are still underdeveloped for the characterization of its interactions with enteropathogens [46].

## Future Perspectives

This section addresses two newly developed techniques that have broad applications, promote intestinal morphology conservation, and grant a higher level of environmental control over explant culture.

**Figure-8:**
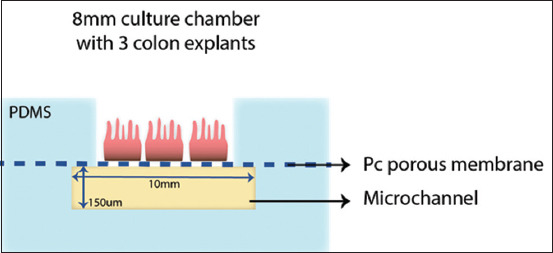
Schematic figure of Baydoun’s interphase microfluidic culture system [47]. Three explants are placed on top of the culture chamber, which is permeated by microchannels conducting the culture media from the inlets to the outlets and providing nutrients through the circular porous membrane. The individual culture apparatus is attached with polydimethylsiloxane, a silicon-like material that holds all elements in place.

**Figure-9:**
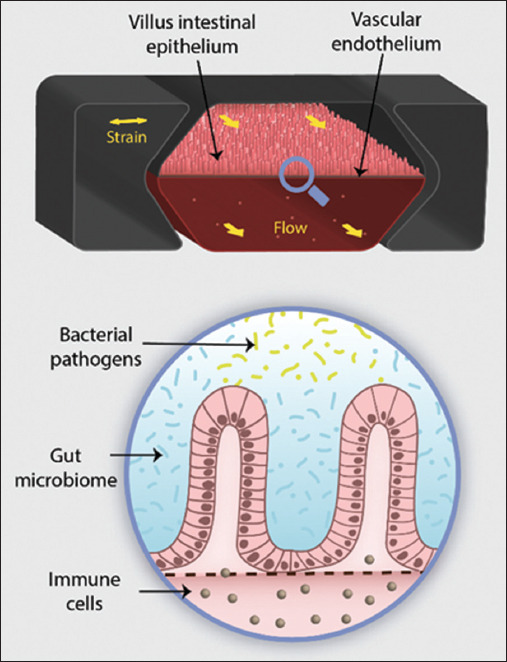
Schematic image of an organ-on-a-chip model [48]. The image displays a culture of intestinal epithelium organized inside the central compartment of the chip. The surface and the basolateral side of the epithelium are separated, and each one receives a different substance (stimulus or culture media) in a constant flow.

The first is the interphase microfluidic culture system proposed by Baydoun *et al*. [27]. The system consists of a central well that relays on top of a porous membrane supported by a microchannel apparatus. It allowed the culture of three explants simultaneously, with no morphological abnormalities for up to 192 h (Figure-8) [47]. The authors claim that improvements must be made to increase the survival rate of explants at the end of the trial. The organ-on-a-chip model is in the early stages of development, but is valuable for allowing the application of desirable cellular, chemical, and mechanical parameters in many types of organs. This unique characteristic can be used, for example, in the analysis of molecular processes concomitant with enteropathies and to assist the development of new therapies (Figure-9) [48].

**Figure-10:**
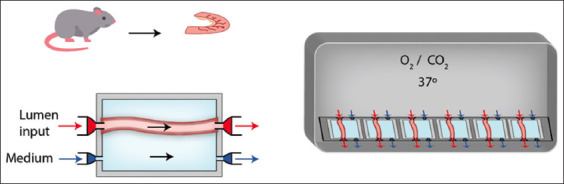
Schematic representation of Yissachar’s setting [49]. (Left) A fragment of the intestine is plugged into an input and an output port responsible for allowing flow transit inside the lumen. The serosal side is also equipped with input and output plugs, which circulate the culture media at the intestine surroundings. (Right) Each set contains six above-described chambers, which are enbibed with a controlled O_2_/CO_2_ atmosphere.

The second is the intestinal gut organ culture system for analyzing host-microbiota interactions developed by Yissachar *et al*. [49]. This model has the characteristic of maintaining the stimulus polarized and preserving the intestinal architecture, allowing the researcher to assess interactions between the microbiome, nervous system, and intestinal immune response (Figure-10) [49]. Because of these characteristics, this system is currently the most sophisticated and accurate method to evaluate interactions between enteropathogens and substances in different species *in vitro*, although it is still non-reproducible due to limitations in replicating the apparatus produced by this research group.

## Conclusion

Intestinal explants cultivated *in vitro* are a powerful and underexplored tool in veterinary research. Its performance is feasible and less costly than *in vivo* experiments and innovative *in vitro* techniques. Following the world’s tendency to reduce the number of animals in research and the great representativeness of physiological features that explants offer, intestinal IVOC methods represent an important tool for animal research to advance the testing of pharmaceutics, microorganisms, and feed additives. This review details key methods for culturing intestinal explants and provides readers with examples of relevant research in which these methods were used. The methods covered are grouped according to their degree of complexity and cost, with the differences in applications and particularities disclosed. By gathering the main technical options and narrowing them based on their suitability, the study of intestinal IVOC methods is facilitated, and their application in veterinary research is more approachable.

## Authors’ Contributions

BRS: Conceptualization of the review, drafted and reviewed the manuscript. RMCG: Supervised and reviewed the manuscript. Both authors have read and approved the final manuscript.
